# The Application of UHPLC-HRMS for Quality Control of Traditional Chinese Medicine

**DOI:** 10.3389/fphar.2022.922488

**Published:** 2022-06-02

**Authors:** Jieyao Ma, Kailin Li, Silin Shi, Jian Li, Sunv Tang, LiangHong Liu

**Affiliations:** ^1^ School of Pharmaceutical Sciences, Hunan Province Key Laboratory for Antibody-Based Drug and Intelligent Delivery System, Hunan University of Medicine, Huaihua, China; ^2^ Hunan Provincial Key Laboratory of Dong Medicine, Hunan University of Medicine, Huaihua, China

**Keywords:** UHPLC-HRMS, traditional Chinese medicine (TCM), structure identification, fingerprint analysis, quality control

## Abstract

UHPLC-HRMS (ultra-high-performance liquid chromatography-high resolution mass spectrometry) is a new technique that unifies the application of UHPLC with HRMS. Because of the high sensitivity and good separation ability of UHPLC and the sensitivity of HRMS, this technique has been widely used for structure identification, quantitative determination, fingerprint analysis, and elucidation of the mechanisms of action of traditional Chinese medicines (TCMs) in recent years. This review mainly outlines the advantages of using UHPLC-HRMS and provides a survey of the research advances on UHPLC-HRMS for the quality control of TCMs.

## Introduction

Traditional Chinese medicine has been used in China for over thousands of years and a long history of development in China and has made great contributions to improving the quality of life and physical health of Chinese people and people around the world. The clinical therapeutic effect of TCM has been documented over the last thousands of years. For example, *Artemisia annua* L is one of most common herbs and is used to treat malignant sores, kill lice, retain the warmth of joints, and improve vision acuity, and has been prescribed in traditional Chinese medical practice for over two thousand years (Tu et al., 2016). Artemisinin was extracted and isolated from *Artemisia annua* L by Tu Youyou in 1971 and was used to successfully treat malaria. Accordingly, Tu Youyou received the 2011 Lasker Award in clinical medicine and shared the 2015 Nobel Prize in Physiology or Medicine with William C. Campbell and Satoshi Ōmura (Efferth et al., 2015). The study on TCM has attracted much attention by researchers worldwide. However, performing quality control of TCM is the only way to ensure its safety and effectiveness and promote the international development of TCM. The current basic mode of quality control of TCM was established by referring to foreign quality control methods for herbal medicines and chemical drugs (Leong et al., 2020). Thin layer chromatography (TLC) and high-performance liquid chromatography (HPLC) play crucial roles in quality control of TCM in the Chinese Pharmacopoeia (ChP). However, the limitations of TLC include the poor repeatability and stability of the results and a mobile phase with high toxicity (Deng et al., 2019). The shortcomings of HPLC include high price of solvents and columns, and a lack of long-term reproducibility due to the proprietary nature of column packing (Siddiqui et al., 2017). In the last decade of the 20th century, liquid chromatography combined with mass spectrometry (LC-MS) was the most important techniques used in the pharmaceutical industry (Niessen et al., 1999).

Nevertheless, the analytical techniques mostly applied for quality control of TCM are high-performance liquid chromatography-ultraviolet-visible detector or photodiode array detector (HPLC-UV/PDA)and/or HPLC-MS. The main drawback is that these techniques are time consuming and do not provide adequate information of resolution of the compounds in TCM. The UHPLC-HRMS system can provide changeable collision energy values and allow the generation of mass information with accuracy and precision, which is ultimately conducive to elucidating the structures and identifying the fragmentation patterns of the compounds of TCM (Lippert et al., 1999).

The aim of this review is to introduce the applications of UHPLC-HRMS using examples of some of the most advanced work in the field of quality control of TCM.

## Advantages of UHPLC-HRMS

UHPLC, as the first producer Waters proclaims, has the advantages of “speed, resolution and sensitivity”. Application of this technique may result in a new direction for liquid chromatography that could reform the field. Compared to traditional chromatographic techniques, such as high-performance thin layer chromatography (HPTLC) and TLC, UHPLC uses column packing material less than 2 μm, and the pressure generated during the use also increases exponentially. Thus, UHPLC has the following unique advantages in the field of quality control of TCM (Khan et al., 2015; Siddiqui et al., 2017; Zhang et al., 2017; Li L. et al., 2020; Kresge et al., 2020):

- Increased selectivity, sensitivty and range of LC analysis.
- The smaller peak width allows identification of a greater number of peaks.
- Fast resolution and quantification of complex components of TCM.
- Withstands the high pressures used in experiments.
- Saves time and solvent.
- Fewer samples are required.
- Increased resolution performance.
- Reduced analysis costs.
- It is an optimal entry point for mass spectrometry detectors.

Without the standard compounds, it is difficult for UHPLC to provide accurate information about the structure of the compounds in TCMs. Meanwhile, a considerable amount of time is required to isolate and purify the compounds of TCMs. With the development of modern analytical techniques, a variety of high resolution mass spectrometry (HRMS) techniques, such as the quadrupole time-of-flight mass spectrometry (Q-TOF-MS) and Q-Exactive Orbitrap mass spectrometry (Q-Orbitrap-MS), have been widely used for the detection and identification of compounds in TCMs (Wang L. et al., 2017; Liang et al., 2021; Tan et al., 2021). Mass spectrometers are more suitable for rapid and cost-efficient analysis and tentative identification of TCMs, and the types that have been used HRMS mass spectrometers (Lv et al., 2018; Li et al., 2021). These mass spectrometers have the advantages such as high mass accuracy, excellent resolution, a fast scan rate, superior sensitivity, and multiple-stage mass spectrometry scanning (MS^n^), and can provide HRMS and MS^n^ data for some compounds present in TCM using a simple data acquisition method (Yang JB. et al., 2019).

## Application of UHPLC-HRMS

### Chemical Characterization of TCM

UHPLC-HRMS is an advanced form of an analytical technique used to separate and identify the complex mixture of components found in TCM to better recognize the role of individual compounds. It is important to make generalizations about fragmentation pathways of reference compounds by the HRMS technique to speculate the identity of potential compounds in TCM.

For example, phenolic acids and prenyl flavonoid glycosides are important constituents of *Epimedii Folium.* An efficient method was developed to enrich these compounds and identify them in *Epimedium koreanum* Nakai (EK) using the UHPLC-HRMS method. Fifty-one prenyl flavonoid glycosides, 18 phenolic acids, and 42 icariin analogues were successfully identified or tentatively identified from *Epimedium koreanum* using this method (Wang et al., 2010; Yang et al., 2017b). Meanwhile, more than 100 compounds were also identified or tentatively identified from *Epimedii Folium* based on the MS/MS of standards, and the compounds were compared with reference results by the UHPLC-HRMS method (Wang Y. et al., 2017; Pilepić et al., 2018; Li M. Y. et al., 2020; Li N. et al., 2020). *Polygonum multiflorum* Thunb. and its processed products have been widely used in China for hundreds of years. *Polygonum multiflorum* exerts liver-tonic and hair-blacking effects. At present, liver injuries caused by taking *P. multiflorum* have been widely reported. To specifically elaborate the bioactive and hepatotoxic constituents, more than 200 compounds, consisting of phenolic acids, flavones, stilbenes, anthraquinones, naphthalenes and their derivatives, were identified or tentatively identified using characteristic diagnostic fragment ions and references based on the UHPLC-HRMS method (Lin et al., 2015; Wang GY. et al., 2017; Yang JB. et al., 2019; Han et al., 2019; Rui et al., 2020).

Using online UHPLC-HRMS, a rapid and credible analytical method was also developed, which was used to identify the chemical conponents of *Polygoni cuspidati* folium and its preparation. Twenty-six chemical constituents, such as derivatives of phenylpropionic acid, tannin, stilbene, flavonoids, torachryson, anthraquinone and their derivatives, were identified or tentatively characterized (Wang X. et al., 2018). *Bupleuri radix* and liquorice are commonly used as medication for protecting liver function. It is well known that the saponins in Bupleuri radix and liquorice can not only promote the metabolism of lipids and sugar, but also exhibit anti-inflammatory function and liver-protective effects. These substances were analyzed by UHPLC-HRMS, and 23 saponins and 9 flavonoid glycosides from liquorice, and 18 saponins from *Bupleuri radix* were tentatively identified based on the characteristic fragment filters and neutral loss filters (Shan et al., 2018). *Schisandra chinensis,* known as WuWeiZi in Chinese, has a good effect in astringing the lung to stoping coughs, arresting sweating, preserving semen and preventing diarrhea. A reliable method was developed for the rapid identification of multiple components in *Schisandra chinensis* by their characteristic fragments and neutral losses using UHPLC-HRMS technology. Accordingly, a total of 30 compounds including 15 lignans, nine triterpenoids, three organic acids and three fatty acids were successfully detected and tentatively identified from *Schisandra chinensis* within 30 min (Yang et al., 2017a). Triterpenoid saponins are the major bioactive constituents of Pulsatilla chinensis. Triterpenoid saponins play important roles in various biological activities such as anti-tumour, cognition-enhancing, anti-biosis, anti-inflammation, hypoglycemia and immunological adjuvant. A systematic strategy based on UHPLC-Q-TOF-MS/MS for the efficient characterisation and identification of triterpenoid saponins in crude extracts from Pulsatilla chinensis has been established (Jin et al., 2018). Bamboo leaves extract (BLE) has a variety of physiological functions such as antitumour, anti-inflammation, antioxidant and hypoglycemic activities and the flavonoids of bamboo leaves are the major active constituents. To profile the flavonoids in the complex BLE, a rapid and sensitive analytical method based on UHPLC-ESI-Q-TOF-MS/MS was developed for the structural identification of the flavonoids in Bambusa chungii leaves extract using accurate mass measurements and characteristic fragmentation patterns (Yuan et al., 2020).

The emergence of UHPLC-HRMS has effectively solved the difficult problems of qualitative analysis of complex medicinal ingredients in TCM. However, the analysis of UHPLC-HRMS also has some deficiencies. Firstly, the volatile components could not identified by UHPLC-HRMS, which was mainly performed by gas chromatography–mass spectrometry (GC-MS). Secondly, qualitative identification of chemical constituents of TCM was detected and identified most combining the MS, MS^2^, chromatography retention time, bibliography data by comparing the mass spectrometry database, performing diagnostic ions filter and molecular network mining. However, the identification of unknown compounds was still inadequate.

### Determination of TCM Components

The UHPLC-HRMS technique has high sensitivity and has been widely used to determine the trace constituents of TCMs.

The dried bark of *Ilex rotunda,* known as Jiubiying, which contains triterpenoids and phenylpropanoids as major bioactive constituents, has been widely applied for clearing heat and removing toxicity in TCM. A validated UHPLC-HRMS method was developed to simultaneously identify and quantify the phenylpropanoids and triterpenoids in the roots, stem bark, stem xylem, leaves, and fruit of *Ilex rotunda*. Meanwhile, the contents of three phenylpropanoids and twelve triterpenoids in the five plant parts were determined with good repeatability, linearity, stability, precision and recovery (Yang et al., 2018). A rapid and reliable UHPLC-ESI-Q-TOF method was also developed to quantify six representative indole alkaloids (vinblastine, vincristine, ajmalicine, vindoline, serpentine, and catharanthine) in *Catharanthus roseus* (Jeong et al., 2018). The radix of *Angelica sinensis* (AS), one of the most important TCMs, is often used for tonifying blood and treatment of amenorrhea and irregular menstruation in females. An UHPLC-HRMS method was established to analyze and identify ferulic acid and phthalides, which could be used to evaluate the quality of AS (Wei et al., 2015a). *Morus alba* L. has an uninterrupted history going back over 4,000 years in China. The leaves of *Morus alba* L have been widely used for the treatment of diabetes as an herbal medicine for thousands of years. An UHPLC-HRMS method was developed for identification and determination of polyhydroxylated alkaloids, such as 1-deoxynojirimycin, with α-glucosidase inhibitor activity in mulberry leaves to evaluate the quality of *Morus alba* L leaves (Ji et al., 2016). *Radix astragali* is one of the most popular TCMs in China due to its effects of invigorating the spleen-stomach and replenishing qi. A UHPLC-HRMS method was used to determine the content of astragaloside I, II and IV in *Radix astragali*, and this method could be effectively applied to evaluate the quality of *Radix astragali* (Han et al., 2016). Poria cocos (Schw.) Wolf has been widely used as a medicine and food in China. An approach based on UHPLC-Q/TOF-MS was utilized to characterize the temporal and spatial variations in the accumulation of specialized metabolites in Fushen, and the quantitative method was successfully applied to simultaneously determine 13 major triterpenoid acids in the nine growth periods and four parts (Yang M. et al., 2021).

### Chemical Fingerprint Analysis

*Phellodendri Amurensis* Cortex (PAC), commonly called Guanhuangbai in ChP, is derived from the dried bark of *Phellodendron amurense* Rupr. Currently, PAC is widely used to treat rheumatoid arthritis, tumors and other diseases. UHPLC/Q-TOF-MS fingerprinting with chemometric methods was first established to identify the major components of Phellodendri amurensis Cortex (PAC). Ten batches of PAC were used to establish the UHPLC/Q-TOF-MS fingerprint. Sixteen common peaks in the fingerprint were obtained, and ten were tentatively identified. The developed fingerprint assay is a powerful method which could be used to conduct quality control of PAC (Li et al., 2010). Ermiao Wan (EW) is a combination formula of *Rhizoma Atractylodis* and *Cortex Phellodendri Chinensis* that is commonly prescribed for patients with gout and hyperuricemia, as described in the ChP (Liu et al., 2006; Yan et al., 2015; Fu et al., 2021; Shan et al., 2021). UHPLC-HRMS fingerprinting combined with the multivariate data mining method (MMA) could provide a validated, rapid and high-throughput methodology for identification of the chemical constituents to ensure the quality of Ermiao Wan (Yan et al., 2015). SiJunZiTang (SJZT), which consists of four herbs, *Radix Ginseng*, *Atractylodes macrocephala*, *Poria cocos* and *Glycyrrhiza uralensis*, is widely used for nourishing qi and to invigorate the spleen and has protective effects on the intestine and stomach against injury, such as stomachache, rugitus, nausea, vomiting or diarrhea (Liu et al., 2006). UHPLC-HRMS fingerprinting provided clues to the contributions of *Atractylodes macrocephala* and *Poria cocos* in SJZT decoction and allowed quality control of SiJunZiTang (SJZT) (Wang Y. et al., 2013). *Gastrodia elata* was first recorded in Shen Nong’s Herbal Medicine Classic and has the functions of calming the liver, spasmolysis and relieving wind in Chinese medicine (Ojemann et al., 2006). The chemical fingerprint of the ethyl acetate fraction of *Gastrodia elata* (EtAcGE) was investigated using UHPLC/HRMS. A total of 38 chemical constituents of EtAcGE were tentatively characterized or identified by comparing the molecular formula and fragment ions with those of known compounds or information available in literatures. The chemical fingerprint and metabolic profile of EtAcGE could provide a basis for the future research on EtAcGE (Tang et al., 2016). *Corydalis yanhusuo* is a well-known Chinese herbal medicine which is frequently used as an analgesic agent in clinics for thousands of years. The *Corydalis yanhusuo-*derived quaternary ammonium alkaloids are effective against myocardial ischemia. The chemical fingerprints of quaternary ammonium alkaloids extracted from the Corydalis yanhusuo speciemens from 37 different sources were identified using UHPLC-HRMS. The fingerprint-efficacy relationship between the chemical fingerprints and cardioprotective effect of Corydalis yanhusuo was investigated (Li et al., 2017). UHPLC-ESI-Q-TOF-MS/MS analysis was conducted to identify the compounds and establish UHPLC fingerprint, explored the bioactive markers of *Codonopsis Radix* (Gao et al., 2019). Zhishi-Xiebai-Guizhi Decoction, has been used for treatment of coronary heart disease and myocardial infarction for nearly two thousand years. An UHPLC-Q-TOF-MS method was utilized for the identification of its multi-constituents, and a total of 148 compounds were identified. In addition, an optimized UHPLC fingerprint analysis, combined with chemometrics was developed for quality assessment (Sang et al., 2020).

### Identification of the Authenticity of TCMs

Donkey-hide gelatin, bovine-hide gelatin, pig-hide gelatin, glue of tortoise shell, equine-hide gelatin and deerhorn glue are widely used as TCMs in China to nourish yin and tonify blood. However, the price varies according to the origins of gelatins. To prevent illegal activities such as manufacturing and marketing counterfeit commodities, a UHPLC-HRMS method combined with a principal component analysis (PCA) was first developed and applied to identify donkey-hide gelatin, bovine-hide gelatin, pig-hide gelatin, tortoise shell glue, equine-hide gelatin and deerhorn glue (Cheng et al., 2012; Jiao et al., 2019). The velvet antler is a non-ossifying hairy young horn derived from the male sika deer (*Cervus nippon* Temminck) or red deer (*Cervus elaphus* Linnaeus) that is used as a medicinal antler in ChP (The State Pharmacopoeia Commission., 2020). Velvet antler has different pharmacological effects on the sexual function and immune system based on its bioactive components and chemical composition (Sui et al., 2014). However, many reindeer antlers are used as medicinal antlers in the market, and the price of reindeer antler is much lower than that of sika deer antler and red deer antler. A UHPLC-HRMS method coupled with principal component analysis (PCA) was successfully developed and used to identify antlers derived from *Cervus elaphus* Linnaeus, *Cervus nippon* Temminck and *Rangifer tarandus* Linnaeus to prevent the sale of counterfeit goods (Guo et al., 2019). *Bletilla striata* has a wide range of applications in pharmacological and cosmetic fields, because of the shortage of resources, there are some substitutes. To distinguish the differences and homologies, a UHPLC fingerprint analysis coupled with chemometric methods were developed for characterization and quality control (Wang et al., 2022). The stems of *Kadsura* interior A. C. Smith has the efficacy of tonifying and invigorating the blood. Its closely related species are morphologically similar, thus likely to exert negative effects on clinical efficacy and clinical medication safety. Combining UHPLC-Q/TOF-MS/MS technique and multivariate data analysis to discover differential metabolites and to comprehensively assesse the chemical constituents, six differential compounds in the stems of K. interior were screened out to distinguish it from K. interior, K. heteroclita, K. longipedunculata, and K. japonica (Xu et al., 2022).

### Identification of Illegal Additives in TCMs

In recent years, the use of TCMs has increased because they are regarded as healthier and safter than chemical drugs and have few side effects (Yuan et al., 2000; Wang J. et al., 2018). Motivated by interest, some unscrupulous manufacturers added chemical ingredients illegally into TCMs, and such behavior could lead to potentially serious public health consequences (Vaclavik et al., 2014). Therefore, it is very important for researchers to develop methods to detect illegal additives to control the quality of TCM.

A UHPLC-HRMS method with an information-dependent acquisition (IDA) mode was developed to screen, identify and quantify the illegal, adulterated, aphrodisiac chemical ingredients in TCMs, and four chemical drugs, including sildenafil, tadalafil, aildenafil and sulfoaildenafil, were detected in some TCMs (Wang XB. et al., 2018). The UHPLC-HRMS method was also applied to identify the illegal additives in TCMs used to relieve cough and asthma or reduce blood glucose. Nine types of chemical medicines including oxytetracycline, sulfamethoxazole, chlorphenamine, diphenhydramine, pentoxyverine, benproperine, prednisone acetate and diazepam were detected in Chinese traditional patent medicines (Chen et al., 2015; Peng et al., 2015). Three types of chemical medicines including phenformin hydrochloride, glibenclamide and pioglitazone hydrochloride were also qualitatively and quantitatively detected in traditional Chinese medicinal preparations (Zhu et al., 2014; Shen et al., 2016). Unauthorized drugs were also found in health foods and herbal products of gout and anti-osteoporosis TCMs. Dexamethasone was detected and confirmed by comparing the MS/MS fragment ion patterns of a reference standard.(Kim et al., 2020). An UHPLC-Q-TOF method was validated for screening, confirmation and quantitation of 31 anti-impotence compounds potentially illegally added to herbal-based dietary supplements. Among 200 batches of herbal-based dietary supplements, sildenafil and/or tadalafil were found to be added illegally in two samples, and low concentration of icariin was detected in one sample (Shi et al., 2020). In general, the UHPLC-HRMS method has been shown to be a powerful tool for routine screening and quantitation of illegal ingredients in TCMs.

### Exploring the Quality-Marker (Q-Marker)

It is well known that TCMs contain various components, and the synergistic effects of these components are conducive to their effectiveness in clinical applications. Meanwhile, one or several chemical markers were previously selected to control the quality of TCM. However, a bottleneck in TCM research has been the low discovery rate of the effective components and the correlation of their effects (Li et al., 2011; Ren et al., 2020). Therefore, Liu *et al.* introduced a new concept of a quality marker (Q-marker) that was used to control the quality of TCMs in 2016 (Liu et al., 2016).

Some TCMs, such as *Lonicera japonica flos* (LJF) and *Lonicera flos* (LF), are easily confused species. It is very difficult to quickly evaluate their potency using conventional methods. UHPLC-HRMS with partial least squares-discriminant analysis (PLS-DA) was used to screen chemical markers for identification of their herbal origin; then, a bioactivity-guided evaluation method was performed to detect the Q-markers. It was found that four NF-kB inhibitors were presented as the representative Q-markers including the following anti-inflammatory compounds: 3, 5-*O*-dicaffeoylquinic acid (3, 5-diCQA), 3-*O*-caffeoylquinic acid (CA), vogeloside and iamarin (Ding et al., 2017). However, some TCMs need to be processed to reduce toxicity and increase efficiency. The underlying mechanism of processing is still not clear, and a Q-marker database for processed TCMs has not been effectively established. For instance, *Radix Wikstroemia indica* (RWI,“Liao Ge Wang” in Chinese) is a type of Chinese herbal medicine (CHM) frequently used in the Miao nationality of South China. RWI is processed by the “sweat soaking method” which effectively decreases its toxicity and preserves its therapeutic effect (Wei et al., 2015b; Chang et al., 2017). Twenty compounds were identified from the ethanol extract of the raw and processed products of RWI based on UHPLC-HRMS, including daphnoretin, emodin, triumbelletin, dibutyl phthalate, and methyl paraben. Three diterpenoids are regarded as the potential Q-markers for quality and safety assessment of the processed RWI by the “sweat soaking method” (Feng et al., 2018). UHPLC-HRMS combined with orthogonal signal correction-partial least squares regression (OSC-PLSR) and the HPLC fingerprint method was applied to search for the Q-markers of Shenzhiling oral liquid, which is used to treat mild to moderate Alzheimer’s disease (AD) (Nie et al., 2016). Sixty-one efficacy-related components were successfully determined by mapping the targets of the disease, and the 3 potential Q-markers of Shenzhiling oral liquid were liquiritin apioside, albiflorin and azelaic acid, which were preliminarily obtained by the above methods (Liu et al., 2019a).

### Identification of Metabolites

Drug metabolism refers to a series of organic reactions after the drugs enter the body, which is also known as biotransformation. For complete knowledge of the therapeutic effectiveness of a TCM, it is essential to identify its metabolites.

For instance, *Scutellaria baicalensis* is one of the most common TCMs and can be used for heat-clearing, dehumidifying and lowering blood sugar (Zhao et al., 2019a; Liao et al., 2021). However, with regard to its main bioactive flavonoids, including baicalein, baicalin, wogonin and wogonoside, the *in vivo* metabolism needs further research (Li et al., 2004). A UHPLC-HRMS technique was used in combination with Metabo-lynx™ software to determine the metabolites and excretion profiles of flavonoids in *S. baicalensis* extract in the feces, urine and bile samples of rats after oral administration of the extract. Nearly 20 metabolites were identified *in vivo*. including glucuronide and sulfate conjugates, and acetylated methylated, deoxygenated and hydroxylated products (Du L.-y. et al., 2015; Du LY. et al., 2015). *Bletilla striata* (Thunb.) Reichb. f (Orchidaceae), also known as Bai-ji, is a type of TCM that is commonly used for the treatment of hematemesis, hemoptysis, traumatic bleeding and other similar disorders (He et al., 2017). A UHPLC-HRMS technique combined with the MS^E^ method was used to identify the metabolic profile of the non-polysaccharide fraction in Sprague-Dawley rats and intestinal bacteria models from *Bletilla striata*. Eight components including 3 metabolites and five prototypes were successfully identified in rat biofluids after oral administration of the non-polysaccharide fraction. The potential metabolic processes, including hydrolysis, deglycosylation, glycosylation, and sulfate conjugation, and pharmacological components could be elaborated using the UHPLC-HRMS method (Yang C. et al., 2019). Tao-Hong-Si-Wu decoction (THSWD) is composed of 6 different TCMs and is widely used for the treatment of blood stasis and gynecologic diseases, such as amenorrhea and dysmenorrhea (Li et al., 2015; Xia et al., 2021). The effect of THSWD on acute blood stasis in rats was systematically studied based on a UHPLC-HRMS metabolomics and a network approach. Fifteen metabolites were screened, and found to be involved ten pathways and five hub metabolites, including L-phenylalanine, L-glutamate, N-acylsphingosine, phosphatidate and arachidonic acid (Ma et al., 2018). Semen Euphorbiae (SE) has significant pharmacological activity. Its toxicity limits its clinical application, and less toxic Semen Euphorbiae Pulveratum (SEP) is often used clinically. a comprehensive metabolomics analysis of serum and urine samples from rats treated with SE and SEP performed by UHPLC/Q-TOF-MS were used to distinguish the differential metabolites of SE and SEP to reveal the metabolic pathways and their significance (Yang Z. et al., 2021).

### Elucidation of the Mechanism of Action of TCMs

TCMs have been used to treat various diseases for thousands of years in Asia and have attracted attention by a growing number of scientists in recent years. However, the mechanisms of action of TCMs are rarely known due to a lack of study by modern scientific methods. The therapeutic efficacy of TCM is usually attributed to the synergistic properties and competitive actions of the TCM formula and its constituents (Zhang et al., 2013).

For example, Taohong Siwu Decoction (TSD), which originated from the “Golden Mirror of Medicine,” can be used to remove blood stasis, promote blood circulation, inhibit inflammatory cytokines and enhance immunity (Duan et al., 2020; Nie et al., 2020). UHPLC-HRMS with PCA and OPLS-DA methods was used to explore changes in the endogenous metabolites, investigate the global alteration of metabolites and evaluate the preventive effect of TSD in rats. The potential metabolic biomarkers of TSD were evaluated using OPLS-DA and a *t*-test, and the mechanisms of action of TSD on acute blood stasis were revealed based on the UHPLC-HRMS platform (Zhang et al., 2018). Sijunzi decoction (SJZD), a classic TCM, has been shown to have therapeutic effects on spleen deficiency syndrome. However, there are few reports on the mechanisms of SJZD in disease treatment. UHPLC-HRMS and PLS-DA methods were also developed to analyze the difference in the global metabolite profile within all groups (untreated rats, normal control rats and SJZD group rats), and variable importance projection (VIP >1) and Student’s t-test (*p* < 0.05) were applied for biomarker selection. Twenty metabolites showed significant differences in the untreated group, and 6 potentially perturbed metabolic pathways were found, which could be conducive to elucidating the mechanism of action of SJZD (Yan et al., 2017). Meanwhile, an online UHPLC-HRMS identification method was employed to examine the synergistic effect of Platycodonis Radix (PG) in the TCM prescription Shengxian Decoction (SXT) (Zhang et al., 2014). In Asia, it is well known that compatibility with TCM can minimize adverse reactions and improve therapeutic efficacy. For instance, UHPLC-HRMS with PCA and OPLS-DA methods could be developed to analyze the attenuation of the toxic effects of an *Aconiti lateralis* radix preparation (named Fuzi in China) and its compatibility with *Glycyrrhizae* radix et rhizome (named Gancao in China). Twelve biomarkers related to Fuzi toxicity and 6 metabolic pathways were identified. With the above methods, the mechanism of compatibility and toxicity attenuation could be evaluated from the perspective of metabolites and could provide a reference for clinical safety (Liu et al., 2019b; Yang B. et al., 2019). *Cantharidin* is the major bioactive component of the blister beetle, which has strong antitumor activity. Clinical application of *cantharidin* is limited because of its toxic effect. An UHPLC-Q-TOF/MS based metabolomics approach in combination with histopathological examination, cell apoptosis assay, and blood biochemical analysis were used to investigate the mechanisms of action of cantharidin-induced hepatotoxicity (Zhu et al., 2019).

### Evaluation of the Quality of TCMs From Different Habitats

Genuine medicinal materials of TCMs refer to those grown in a specific area with high quality and effectiveness. Due to the influence of growing conditions, climate and other factors, analysis of the characteristic constituents of TCM has shown differences in TCMs from different habitats.

For example, *Atractylodis Macrocephalae Rhizoma* (AMR) was widely used to reinforce the spleen, nourish Qi and remove dampness in Chinese medicine and is mainly distributed in Anhui, Jiangxi, Henan, Hebei, Hubei, Zhejiang and other areas. To evaluate the quality of AMR from genuine producing areas (Zhejiang) and other regions, a UHPLC-HRMS technique combined with multivariate statistical analysis was developed to investigate the common and different components of AMR from different regions. Sixteen major differential compounds, such as tyrosine, methylated atractylenolide I, atractylenolide I, II and III, and atractylone were selected from samples of 7 different regions. Because AMR from Zhejiang shows better results than samples from other regions, tyrosine, and dehydroaromadendrene could be used as the index ingredients for evaluation of the genuineness of AMP based on the above methods (Huang et al., 2017). *Moutan Cortex*, the root cortex of *Paeonia suffruticosa* Andr., is an important TCM that is used as an analgesic, antispasmodic and anti-inflammatory agent (Zhang et al., 2020). Based on its origin, it can be classified as a genuine medicinal material, Fengdanpi (Tonglin, Anhui), or general herb (other areas). The UHPLC-HRMS technique combined with the PCA, PLS-DA, and OPLS-DA methods was used to identify and analyze the common and different components of *Moutan Cortex*. The medicinal materials from different origins were clustered into different groups by PCA. Five biomarkers were successfully obtained by the UHPLC-HRMS, PLS-DA and OPLS-DA methods. These could provide a theoretical basis for understanding the chemical material composition and quality evaluation of *Moutan Cortex* (Hu et al., 2016). *Ophiopogonis radix*, the tuberous root of Ophiopogon japonicas (Thunb.) Ker-Gawl (Liliaceae), known as Maidong in Chinese, is a common TCM that is used to alleviate symptoms of diabetes and cardiovascular diseases (Wang et al., 2019). Multiple bioactive constituents analysis based on the UHPLC-HRMS technique combined with multivariate statistical analysis was used to evaluate the effects of two types of *Ophiopogonis radix* that originated form Hangzhou and Sichuang. The results showed that the quality of *Ophiopogonis radix* from genuine producing areas (Sichuan) was better than that from other areas (Zhejiang) (Tan et al., 2019). *Sophora tonkinensis* is widely used as TCM for treating the swelling of the gums, tongue and mouth sores due to flaring up of stomach fire. Alkaloids are the major bioactive components. UHPLC-Q-TOF-MS/MS was applied in identifying and characterizing alkaloids in *S. tonkinensis* root of two different habitats. The Radix *Sophora Tonkinensis* for Guozhou, Sichuan province have difference by comparative analysis of alkaloids for two different habitats (Zong et al., 2022). UHPLC-Q-TOF/MS was also used to compare the differences of *Paeonia lactiflora* from different habitats, Sichuan, Hebei, Henan, Shanxi and Anhui. (Zhao et al., 2019b).

### Others

*Cordyceps sinensis* (Berk.) Sacc. is a well-known TCM and has many active ingredients, such as polysaccharides, nucleosides, cordycepic acid, and sterols (Wang Z. et al., 2013; Xia et al., 2022; Yuan et al., 2022). *C. sinensis* (Berk.) Sacc. has been commonly used for the treatment of night sweats, hyperlipidemia, hyperglycemia, respiratory disease, renal dysfunction and failure, arrhythmias, other heart disease, and liver disease. To examine the correlation between the quality and chemical constituents of different parts of *C. sinensis* (Berk.) Sacc., UHPLC-HRMS with the PCA and PLS-DA methods was used to analyze the chemical constituents of three parts of Cordyceps including the seats, heads and insect worms. Eleven differentiated compounds were identified from Cordyceps seats, heads and insect worms and mainly consisted of fatty acids and their derivatives (Qin et al., 2018). The established method could provide a scientific reference for clarifying the pharmacodynamics and the mechanism of quality assessment of *C. sinensis* (Berk.) Sacc. and provide a foundation for its rapid identification, quality control and utilization of *C. sinensis* (Berk.) Sacc. Meanwhile, the UHPLC-HRMS technique could be applied to pharmacokinetic studies in rats or humans. For example, this method could be used to determine the content of tumulosis acid and dehydro-tumulosic acid in rat plasma after oral administration of Poria triterpenoid extract powder and soft capsules because different dosage forms could affect the bioavailability of these compounds (Wen et al., 2017).

## Conclusion

The UHPLC-HRMS technique is widely used for applications in chromatography-mass spectrometric analysis. This technique provides not only rapid and improved chromatographic separation and short chromatographic run time, but also high sensitivity and selectivity, accurate measurement, and reliable chemical fragmentation, that are ultimately helpful for elucidating the structure of various compounds. This technique could be applied to obtain better results than conventional TLC, HPLC-UV and HPLC-MS techniques. In this review, we go over the advantages and applications of UHPLC-HRMS in the analysis of TCM constituents, and examples are provided that focus on the difficult points mentioned above. Attention should be paid to the accuracy of qualitative analysis when applying this method for the quality control of TCM. The mass spectrometry-based natural product database should be a feasible way to identify chemicalls easily, effectively and accurately. The utilization of artificial intelligence in data mining and interpretation will be an effective method.

In summary, UHPLC-HRMS is a very useful tool for the determination of components or metabolites of TCMs that might link to various diseases. UHPLC-HRMS is a simple and rapid method for the quality control of TCMs. It is important to build a mass spectrum database and utilize the artificial intelligence to identify the complex substances of TCMs accurately and establish a reasonable quality appraising standard for TCMs.

## Abbreviations

UHPLC-HRMS, Ultra-high-performance liquid chromatography-high resolution mass spectrometry; TCMs, Traditional Chinese medicines; TLC, Thin layer chromatography; HPTLC, High-performance thin layer chromatography; LC-MS, Liquid chromatography combined with mass spectrometry; HPLC-UV/PDA, High-performance liquid chromatography-ultraviolet-visible detector or photodiode array detector; Q-TOF-MS, Quadrupole time-of-flight mass spectrometry; UHPLC/Q-TOF-MS, Ultr-high performance liquid chromatography coupled to Quadrupole time-of-flight mass spectrometry; Q-Orbitrap-MS, Q-Exactive Orbitrap mass spectrometry; UHPLC-ESI-Q-TOF, Ultra-high performance liquid chromatographic electrospray ionization quadrupole- time of flight mass spectrometry; ChP, Chinese Pharmacopoeia; PCA, Principal component analysis.

## References

[B1] Chang H. Wang Y. Gao X. Song Z. Awale S. Han N. (2017). Lignans from the Root of Wikstroemia Indica and Their Cytotoxic Activity Against PANC-1 Human Pancreatic Cancer Cells. Fitoterapia 121, 31–37. 10.1016/j.fitote.2017.06.012 28629933

[B2] Chen X. H. Qin J. Su J. Ren X. Y. Zeng L. G. Kuang G. (2015). Determination of 8 Kinds of Chemical Medicines Illegally Added in Traditional Chinese Medicines for Relieving Cough and Asthma by UPLC-Q-TOF. Chin. J. Exp. Traditional Med. Formulae 21 (04), 64–67. 10.13422/j.cnki.syfjx.2015040064

[B3] Cheng X. L. Wei F. Xiao X. Y. Zhao Y. Y. Shi Y. Liu W. (2012). Identification of Five Gelatins by Ultra Performance Liquid Chromatography/Time-Of-Flight Mass Spectrometry (UPLC/Q-TOF-MS) Using Principal Component Analysis. J. Pharm. Biomed. Anal. 62, 191–195. 10.1016/j.jpba.2011.12.024 22257536

[B4] Deng Z. Jing W. G. Liu A. (2019). Discussion about Application of Thin Layer Chromatography in Current Quality Standard Control. Chin. J. Exp. Traditional Med. Formulae 25, 201–206. 10.13422/j.cnki.syfjx.20190202

[B5] Ding G. Wang Y. Liu A. Hou Y. Zhang T. Bai G. (2017). From Chemical Markers to Quality Markers: An Integrated Approach of UPLC/Q-TOF, NIRS, and Chemometrics for the Quality Assessment of Honeysuckle Buds. RSC Adv. 7 (36), 22034–22044. 10.1039/C6RA28152D

[B6] Du L.-y. Qian D.-w. Shang E.-x. Jiang S. Liu P. Guo J.-m. 2015a). UPLC-MS Based Metabolite Profiles of Two Major Bioactive Components in Herb Pair Scutellaria-Coptis Metabolized by Intestinal Bacteria Derived from Healthy Rats and Rats with Type 2 Diabetes. Anal. Methods. 7(13), 5574–5582. 10.1039/C5AY00931F

[B7] Du L. Y. Qian D. W. Shang E. X. Liu P. Jiang S. Guo J. M. (2015b). UPLC-Q-TOF/MS-Based Screening and Identification of the Main Flavonoids and Their Metabolites in Rat Bile, Urine and Feces After Oral Administration of Scutellaria Baicalensis Extract. J. Ethnopharmacol. 169, 156–162. 10.1016/j.jep.2015.04.039 25926286

[B8] Duan X. Pan L. Peng D. Bao Q. Xiao L. Zhou A. (2020). Analysis of the Active Components and Metabolites of Taohong Siwu Decoction by Using Ultra High Performance Liquid Chromatography Quadrupole Time‐of‐Flight Mass Spectrometry. J. Sep. Sci. 43, 4131–4147. 10.1002/jssc.202000498 32914552

[B9] Efferth T. Zacchino S. Georgiev M. I. Liu L. Wagner H. Panossian A. (2015). Nobel Prize for Artemisinin Brings Phytotherapy into the Spotlight. Phytomedicine 22 (13), A1–A3. 10.1016/j.phymed.2015.10.003 26563851

[B10] Feng G. Chen Y.-l. Li W. Li L.-l. Wu Z.-g. Wu Z.-j. (2018). Exploring the Q-Marker of “sweat Soaking Method” Processed Radix Wikstroemia Indica: Based on the “Effect-Toxicity-Chemicals” Study. Phytomedicine 45, 49–58. 10.1016/j.phymed.2018.03.063 29691116

[B11] Fu X. L. Zhou J. Tang W. W. Liu Y. Li Z. L. Li P. (2021). Study on the Compatibility Effect and Active Constituents of Atractylodis Rhizoma in Ermiao Wan Against Acute Gouty Arthritis. J. Ethnopharmacol. 279, 114353. 10.1016/j.jep.2021.114353 34161798

[B12] Gao S. Liu J. Wang M. Liu Y. Meng X. Zhang T. (2019). Exploring on the Bioactive Markers of Codonopsis Radix by Correlation Analysis Between Chemical Constituents and Pharmacological Effects. J. Ethnopharmacol. 236, 31–41. 10.1016/j.jep.2019.02.032 30776470

[B13] Guo X. H. Cheng X. L. Liu W. X. Li M. H. Wei F. Ma S. C. (2019). Identification of Velvet Antler and its Mixed Varieties by UPLC-QTOF-MS Combined with Principal Component Analysis. J. Pharm. Biomed. Anal. 165, 18–23. 10.1016/j.jpba.2018.10.009 30500596

[B14] Han L. Wang P. Wang Y. Zhao Q. Zheng F. Dou Z. (2019). Rapid Discovery of the Potential Toxic Compounds in Polygonum Multiflorum by UHPLC/Q-Orbitrap-MS-Based Metabolomics and Correlation Analysis. Front. Pharmacol. 10, 329. 10.3389/fphar.2019.00329 31057397 PMC6477936

[B15] Han X. Y. Peng B. Wang H. Zhang C. Zeng Z. P. (2016). UPLC/Q-TOF-MS-Based Chemical Profiling Approach in Studying the Effects of Amic Solution Hydrolysis on Extraction of Radix Astragali. World Chin. Med. 11 (03), 523–528+532. 10.3969/j.issn.1673-7202.2016.03.040

[B16] He X. Wang X. Fang J. Zhao Z. Huang L. Guo H. (2017). Bletilla Striata: Medicinal Uses, Phytochemistry and Pharmacological Activities. J. Ethnopharmacol. 195, 20–38. 10.1016/j.jep.2016.11.026 27865796

[B17] Hu Y. F. Pei Y. M. Wu H. Xu Q. Xu G. B. Jiang L. (2016). Difference Analysis of Chemical Compositions in Moutan Cortex from Different Origins by UPLC-Q-TOF-MS. Chin. Traditional Herb. Drugs 47, 2984–2992. 10.7501/j.issn.0253-2670.2016.17.005

[B18] Huang X. F. OuYang H. Li J. M. Lu Y. J. Li W. Gong Q. F. (2017). Identification of Characteristic Constituents in Atractylodis Macrocephalae Rhizoma from Different Regions by UPLC-Q-TOF-MS/MS. Chin. J. Exp. Traditional Med. Formulae 23, 27–33. 10.13422/j.cnki.syfjx.201720027

[B19] Jeong W. T. Lim H. B. (2018). A UPLC-ESI-Q-TOF Method for Rapid and Reliable Identification and Quantification of Major Indole Alkaloids in Catharanthus Roseus. J. Chromatogr. B Anal. Technol. Biomed. Life Sci. 1080, 27–36. 10.1016/j.jchromb.2018.02.018 29477064

[B20] Jiao Y. Wang B. Zhou Q. Q. Hang B. J. Lin Y. Q. (2019). Petection of Equine-Hide Gelatin in Ejiao by UPLC-MS. Chin. J. Pharm. Anal. 39 (5), 864–869. 10.16155/j.0254-1793.2019.05.14

[B21] Jin M. M. Zhang W. D. Jiang H. H. Du Y. F. Guo W. Cao L. (2018). UPLC-Q-TOF-MS/MS-Guided Dereplication of Pulsatilla Chinensis to Identify Triterpenoid Saponins. Phytochem. Anal. 29 (5), 516–527. 10.1002/pca.2762 29637651

[B22] Khan H. Ali J. (2015). UHPLC/Q-ToF-MS Technique: Introduction and Applications. Loc 12 (6), 371–378. 10.2174/1570178612666150331204147

[B23] Kim N. S. Kim J. Lim N. Y. Lee J. H. Park S. Kang H. (2020). Simultaneous Determination of Illegal Drug Substances in Dietary Supplements for Gout and Osteoporosis Using Ultra-Performance Liquid Chromatography and Liquid Chromatography-Quadrupole-Time-Of-Flight Mass Spectrometry. J. Pharm. Biomed. Anal. 179, 113003. 10.1016/j.jpba.2019.113003 31816474

[B24] Kresge G. A. Grosse S. Zimmer A. Grinias K. M. De Pra M. Wong J. T. 2020). Strategies in Developing High-Throughput Liquid Chromatography Protocols for Method Qualification of Pharmacopeial Monographs. J. Sep. Sci. 43(15), 2964–2970. 10.1002/jssc.202000403 32388922

[B25] Leong F. Hua X. Wang M. Chen T. Song Y. Tu P. (2020). Quality Standard of Traditional Chinese Medicines: Comparison between European Pharmacopoeia and Chinese Pharmacopoeia and Recent Advances. Chin. Med. 15(1), 76–20. 10.1186/s13020-020-00357-3 32742301 PMC7388521

[B26] Li H. B. Jiang Y. Chen F. (2004). Separation Methods Used for Scutellaria Baicalensis Active Components. J. Chromatogr. B Anal. Technol. Biomed. Life Sci. 812, 277–290. 10.1016/j.jchromb.2004.06.045 PMC7105199 15556504

[B27] Li L. Wang Y. Liu S. (2020c). Application of Pseudotargeted Method Combined with Multivariate Statistical Analysis for the Quality Assessment of Traditional Chinese Medicine Preparation, Sanhuang Tablet as a Case. Anal. Bioanal. Chem. 412(23), 5863–5872. 10.1007/s00216-020-02813-3 32686055

[B28] Li L. Yang N. Nin L. Zhao Z. Chen L. Yu J. (2015). Chinese Herbal Medicine Formula Tao Hong Si Wu Decoction Protects Against Cerebral Ischemia-Reperfusion Injury via PI3K/Akt and the Nrf2 Signaling Pathway. J. Nat. Med. 69 (1), 76–85. 10.1007/s11418-014-0865-5 25149059

[B29] Li M. Y. Sun E. Xu F. J. Xu J. D. Jia X. B. (2020a). Analysis Changes of Epimedii Folium's Flavonoids Before and After Processing Based on UPLC-Q/TOF-MS. Chin. Traditional Herb. Drugs 51 (11), 2900–2907. 10.7501/j.issn.0253-2670.2020.11.007

[B30] Li N. Xie L. Yang N. Sun G. Liu H. Bi C. (2020b). Rapid Classification and Identification of Chemical Constituents in Epimedium Koreanum Nakai by UPLC-Q-TOF-MS Combined with Data Post-Processing Techniques. Phytochem. Anal. 32 (4), 575–591. 10.1002/pca.3007 33167069

[B31] Li Q. Guan H. Wang X. He Y. Sun H. Tan W. (2017). Fingerprint-Efficacy Study of the Quaternary Alkaloids in Corydalis Yanhusuo. J. Ethnopharmacol. 207, 108–117. 10.1016/j.jep.2017.06.036 28647508

[B32] Li R. Wei M. Guo G. Li Y. Pan X. Song X. (2021). Analysis of Main Components in Jujube and Mulberry Extracts by High-Sensitive HPLC-ESI-Q-TOF-MS/MS. J. Chromatogr. Sci. 59 (9), 806–812. 10.1093/chromsci/bmaa133 33598689

[B33] Li S. P. Zhao J. Yang B. (2011). Strategies for Quality Control of Chinese Medicines. J. Pharm. Biomed. Anal. 55 (4), 802–809. 10.1016/j.jpba.2010.12.011 21215546

[B34] Li Y. Zhang T. Zhang X. Xu H. Liu C. (2010). Chemical Fingerprint Analysis of Phellodendri Amurensis Cortex by Ultra Performance LC/Q-TOF-MS Methods Combined with Chemometrics. J. Sep. Sci. 33 (21), 3347–3353. 10.1002/jssc.201000426 21049522

[B35] Liang G. Yang J. Liu T. Wang S. Wen Y. Han C. (2021). A Multi-Strategy Platform for Quality Control and Q-Markers Screen of Chaiqin Chengqi Decoction. Phytomedicine 85, 153525. 10.1016/j.phymed.2021.153525 33740732

[B36] Liao H. Ye J. Gao L. Liu Y. (2021). The Main Bioactive Compounds of Scutellaria Baicalensis Georgi. For Alleviation of Inflammatory Cytokines: A Comprehensive Review. Biomed. Pharmacother. 133, 110917. 10.1016/j.biopha.2020.110917 33217688

[B37] Lin L. F. Ni B. Lin H. Zhang M. Yan L. Qu C. (2015). Simultaneous Determination of 14 Constituents of Radix Polygoni Multiflori from Different Geographical Areas by Liquid Chromatography-Tandem Mass Spectrometry. Biomed. Chromatogr. 29, 1048–1055. 10.11669/cpj.2015.12.01310.1002/bmc.3391 25450501

[B38] Lippert J. A. Xin B. Wu N. Lee M. L. (1999). Fast Ultrahigh-Pressure Liquid Chromatography: On-Column UV and Time-Of-Flight Mass Spectrometric Detection. J. Micro. Sep. 11 (9), 631–643. 10.1002/(SICI)1520-667X(199911)11:93.0.CO;2-I10.1002/(sici)1520-667x(199911)11:9<631:aid-mcs1>3.0.co;2-i

[B39] Liu Y. Wei M. Yue K. Wang R. Ma Y. Men L. (2019b). Non-Target Metabonomic Method Provided New Insights on the Therapeutical Mechanism of Gancao Fuzi Decoction on Rheumatoid Arthritis Rats. J. Chromatogr. B Anal. Technol. Biomed. Life Sci. 1105, 93–103. 10.1016/j.jchromb.2018.11.015 30576890

[B40] Liu Y. Yang J. Cai Z. (2006). Chemical Investigation on Sijunzi Decoction and its Two Major Herbs Panax Ginseng and Glycyrrhiza Uralensis by LC/MS/MS. J. Pharm. Biomed. Anal. 41 (5), 1642–1647. 10.1016/j.jpba.2006.02.033 16574366

[B41] Liu C. X. Chen S. L. Xiao X. H. Zhang T. J. Hou W. B. Liao M. L. (2016). A New Concept on Quality Marker of Chinese Materia Medica: Quality Control for Chinese Medicinal Products. Chin. Traditional Herb. Drugs, 1443–1457. 10.7501/j.issn.0253-2670.2016.09.001

[B42] Liu X. Y. Jiang W. W. Jiang H. Q. Su M. Sun Y. Zang H. C. (2019a). Preliminary Discovery of Quality Marker (Q-Marker) of Shenzhiling Oral Liquid Based on “Fingerprint-Efficacy-Pharmacokinetics” Correlation. Chin. Traditional Herb. Drugs 50, 4603–4612. 10.7501/j.issn.0253-2670.2019.19.012

[B43] Lv X. J. Sun Z. Wang P. L. Yang J. Xu T. Y. Jia Q. Q. (2018). Chemical Profiling and Quantification of Dan-Deng-Tong-Nao-Capsule Using Ultra High Performance Liquid Chromatography Coupled with High Resolution Hybrid Quadruple-Orbitrap Mass Spectrometry. J. Pharm. Biomed. Anal. 148, 189–204. 10.1016/j.jpba.2017.09.034 29040936

[B44] Ma Q. Li P. L. Hua Y. L. Ji P. Yao W. L. Zhang X. S. (2018). Effects of Tao-Hong-Si-Wu Decoction on Acute Blood Stasis in Rats Based on a LC-Q/TOF-MS Metabolomics and Network Approach. Biomed. Chromatogr. 32 (4), e4144. 10.1002/bmc.4144 29149492

[B45] Nie H. L. Zhang D. L. Nie L. Zang H. C. Zeng Y. Z. (2016). HPLC Fingerprint Study and Quality Evaluation of Shenzhiling Oral Solution. J. Pharmaceutical Res. 35 (07), 386–389. 10.13506/j.cnki.jpr.2016.07.005

[B46] Nie X. Cheng Y. F. Wang L. Fu C. M. He Y. Zhang J. M. (2020). Review of Chemical Constituents, Pharmacological Effects and Clinical Applications of Taohong Siwutang and Predictive Analysis of its Quality Marke. Chin. J. Exp. Traditional Med. Formula 26 (04), 226–234. 10.13422/j.cnki.syfjx.20191953

[B47] Niessen W. M. (1999). State-of-the-Art in Liquid Chromatography-Mass Spectrometry. J. Chromatogr. A 856 (1-2), 179–197. 10.1016/S0021-9673(99)00480-X 10526788

[B48] Ojemann L. M. Nelson W. L. Shin D. S. Rowe A. O. Buchanan R. A. (2006). Tian Ma, an Ancient Chinese Herb, Offers New Options for the Treatment of Epilepsy and Other Conditions. Epilepsy Behav. 8 (2), 376–383. 10.1016/j.yebeh.2005.12.009 16461011

[B49] Peng Y. W. Shen L. H. Wang L. Jin J. Yang M. Z. (2015). Detection of Oxytetracycline, Chlorpheniramine Maleate and Prednisone Acetate in Two Chinese Traditional Patent Medicines by UPLC/Q-TOF-MS. Chin. Tradit. Pat. Med. 37 (09), 1959–1964. 10.3969/j.issn.1001-1528.2015.09.019

[B50] Pilepić K. H. Yang Z. Chen J. Chen X. Wang Y. Zhao J. (2018). Flavonoids in Natural and Tissue Cultured Materials of Epimedium Alpinum Identified by Using UHPLC-Q-TOF-MS/MS. Int. J. Mass Spectrom. 434, 222–232.

[B51] Qin W. H. Hua L. Guo Y. L. Wang Y. H. Ran J. C. Yang Y. (2018). Differentiation of Different Parts of Cordyceps Sinensis Based on UPLC- Q- TOF- MS Combined with Metabolomics Methods. Chin. J. Exp. Traditional Med. Formulae 24 (21), 69–76. 10.13422/j.cnki.syfjx.20181910

[B52] Ren J. L. Zhang A. H. Kong L. Han Y. Yan G. L. Sun H. (2020). Analytical Strategies for the Discovery and Validation of Quality-Markers of Traditional Chinese Medicine. Phytomedicine 67, 153165. 10.1016/j.phymed.2019.153165 31954259

[B53] Rui W. Xia W. Zhao W. Li B. Li J. Feng Y. (2020). Differential Constituents in Roots, Stems and Leaves of Polygonum Multiflorum Thunb. Screened by UPLC/ESI-Q-TOF-MS and Multivariate Statistical Analysis. J. Chromatogr. Sci. 58 (2), 136–143. 10.1093/chromsci/bmz086 31746330

[B54] Sang Q. Jia Q. Zhang H. Lin C. Zhao X. Zhang M. (2020). Chemical Profiling and Quality Evaluation of Zhishi-Xiebai-Guizhi Decoction by UPLC-Q-TOF-MS and UPLC Fingerprint. J. Pharm. Biomed. Anal. 194, 113771. 10.1016/j.jpba.2020.113771 33280997

[B55] Shan B. Chen T. Huang B. Liu Y. Chen J. (2021). Untargeted Metabolomics Reveal the Therapeutic Effects of Ermiao Wan Categorized Formulas on Rats with Hyperuricemia. J. Ethnopharmacol. 281, 114545. 10.1016/j.jep.2021.114545 34419610

[B56] Shan L. Yang N. Zhao Y. Sheng X. Yang S. Li Y. (2018). A Rapid Classification and Identification Method Applied to the Analysis of Glycosides in Bupleuri Radix and Liquorice by Ultra High Performance Liquid Chromatography Coupled with Quadrupole Time-Of-Flight Mass Spectrometry. J. Sep. Sci. 41 (19), 3791–3805. 10.1002/jssc.201800619 30074686

[B57] Shen L. H. Peng Y. W. Chen G. Q. (2016). Rapid Detection of Phenformin Hydrochloride, Glibenclamide and Pioglitazone Hydrochloride Illegally Mixed in Traditional Chinese Medicinal Preparation for Antidiabetics by UPLC-Q-TOF-MS. Chin. J. Mod. Appl. Pharm. 33 (09), 1160–1165. 10.13748/j.cnki.issn1007-7693.2016.09.016

[B58] Shi S. Wu Y. Zhou M. Cheng Q. (2020). Simultaneous Analysis of 31 Anti-impotence Compounds Potentially Illegally Added to Herbal-Based Dietary Supplements by Ultra-High-Performance Liquid Chromatography Coupled with Quadrupole Time-Of-Flight Mass Spectrometry. J. Chromatogr. B Anal. Technol. Biomed. Life Sci. 1144, 122077. 10.1016/j.jchromb.2020.122077 32251992

[B59] Siddiqui M. R. AlOthman Z. A. Rahman N. (2017). Analytical Techniques in Pharmaceutical Analysis: A Review. Arabian J. Chem. 10, S1409–S1421. 10.1016/j.arabjc.2013.04.016

[B60] Sui Z. Zhang L. Huo Y. Zhang Y. (2014). Bioactive Components of Velvet Antlers and Their Pharmacological Properties. J. Pharm. Biomed. Anal. 87, 229–240. 10.1016/j.jpba.2013.07.044 24029381

[B61] Tan M. Chen J. Wang C. Zou L. Chen S. Shi J. (2019). Quality Evaluation of Ophiopogonis Radix from Two Different Producing Areas. Molecules 24 (18), 3220. 10.3390/molecules24183220 PMC6766908 31487946

[B62] Tan X. M. Li Q. Wang Y. D. Wang T. L. Yang J. Sun B. D. (2021). UPLC‐Q‐TOF‐MS/MS Analysis of the Guaiane Sesquiterpenoids Oxytropiols A-J and Detection of Undescribed Analogues from the Locoweed Endophytic Fungus Alternaria Oxytropis (Pleosporaceae). Phytochem. Anal. 33, 344–354. 10.1002/pca.3092 34755399

[B63] Tang C. Wang L. Liu X. Cheng M. Xiao H. (2016). Chemical Fingerprint and Metabolic Profile Analysis of Ethyl Acetate Fraction of Gastrodia Elata by Ultra Performance Liquid Chromatography/quadrupole-Time of Flight Mass Spectrometry. J. Chromatogr. B Anal. Technol. Biomed. Life Sci. 1011, 233–239. 10.1016/j.jchromb.2015.09.043 26621783

[B64] The State Pharmacopoeia Commission (2020). The People’s Republic of China Pharmacopoeia. Beijing: Chinese Medicine Science and Technology Press, 336–337.

[B65] Tu Y. (2016). Artemisinin-a Gift from Traditional Chinese Medicine to the World (Nobel Lecture). Angew. Chem. Int. Ed. Engl. 55 (35), 10210–10226. 10.1002/anie.201601967 27488942

[B66] Vaclavik L. Krynitsky A. J. Rader J. I. (2014). Targeted Analysis of Multiple Pharmaceuticals, Plant Toxins and Other Secondary Metabolites in Herbal Dietary Supplements by Ultra-High Performance Liquid Chromatography-Quadrupole-Orbital Ion Trap Mass Spectrometry. Anal. Chim. Acta 810, 45–60. 10.1016/j.aca.2013.12.006 24439505

[B67] Wang G. Y. Shang J. Wu Y. Ding G. Xiao W. (2017b). Rapid Characterization of the Major Chemical Constituents from Polygoni Multiflori Caulis by Liquid Chromatography Tandem Mass Spectrometry and Comparative Analysis with Polygoni Multiflori Radix. J. Sep. Sci. 40 (10), 2107–2116. 10.1002/jssc.201601255 28322504

[B68] Wang H. Y. Wang C. Guo S. C. Chen Z. C. Peng Z. T. Duan R. (2019). Polysaccharide Deriving from Ophiopogonis Radix Promotes Metabolism of Ginsenosides in the Present of Human Gut Microbiota Based on UPLC-MS/MS Assay. J. Pharm. Biomed. Anal. 175, 112779. 10.1016/j.jpba.2019.112779 31349212

[B69] Wang J. Wong Y. K. Liao F. (2018b). What Has Traditional Chinese Medicine Delivered for Modern Medicine? Expert Rev. Mol. Med. 20, e4. 10.1017/erm.2018.3 29747718

[B70] Wang L. Sang M. Liu E. Banahene P. O. Zhang Y. Wang T. (2017a). Rapid Profiling and Pharmacokinetic Studies of Major Compounds in Crude Extract from Polygonum Multiflorum by UHPLC-Q-TOF-MS and UPLC-MS/MS. J. Pharm. Biomed. Anal. 140, 45–61. 10.1016/j.jpba.2017.03.016 28342304

[B71] Wang R. Qin Y. Zhou J. Wang J. Shu H. Zhou S. (2022). Comprehensive Evaluation of Bletilla Striata and its Substitutes by Combining Phenotypic Characteristic, Chemical Composition, and Anti-Melanogenic Activity. Phytochemistry 195, 113059. 10.1016/j.phytochem.2021.113059 34933209

[B72] Wang X. Qin Y. Li G. Q. Chen S. Ma J. Q. Guo Y. L. (2018a). Study on Chemical Constituents in Polygoni Cuspidati Folium and its Preparation by UPLC-ESI-Q-TOF-MS/MS. J. Chromatogr. Sci. 56 (5), 425–435. 10.1093/chromsci/bmy017 29554228

[B73] Wang X. B. Zheng J. Li J. J. Yu H. Y. Li Q. Y. Xu L. H. (2018c). Simultaneous Analysis of 23 Illegal Adulterated Aphrodisiac Chemical Ingredients in Health Foods and Chinese Traditional Patent Medicines by Ultrahigh Performance Liquid Chromatography Coupled with Quadrupole Time-Of-Flight Mass Spectrometry. J. Food Drug Anal. 26 (3), 1138–1153. 10.1016/j.jfda.2018.02.003 29976406 PMC9303026

[B74] Wang Y. Guo Z. Jin Y. Zhang X. Wang L. Xue X. (2010). Identification of Prenyl Flavonoid Glycosides and Phenolic Acids in Epimedium Koreanum Nakai by Q-TOF-MS Combined with Selective Enrichment on "click Oligo (Ethylene Glycol)" Column. J. Pharm. Biomed. Anal. 51 (3), 606–616. 10.1016/j.jpba.2009.09.033 19854603

[B75] Wang Y. He S. Cheng X. Lu Y. Zou Y. Zhang Q. (2013a). UPLC-Q-TOF-MS/MS Fingerprinting of Traditional Chinese Formula SiJunZiTang. J. Pharm. Biomed. Anal. 80, 24–33. 10.1016/j.jpba.2013.02.021 23511229

[B76] Wang Y. Yuan L. Li Y. B. Zhang Y. J. (2017c). Analysis on Chemical Constituents of Epimedii Folium by UPLC-Q-TOF-MS. Chin. Traditional Herb. Drugs, 2625–2631. 10.7501/j.issn.0253-2670.2017.13.007

[B77] Wang Z. Li N. Wang M. Wang Y. Du L. Ji X. (2013b). Simultaneous Determination of Nucleosides and Their Bases in Cordyceps Sinensis and its Substitutes by Matrix Solid-Phase Dispersion Extraction and HPLC. J. Sep. Sci. 36 (14), 2348–2357. 10.1002/jssc.201300204 23677705

[B78] Wei L. Wang X. Mu S. Sun L. Yu Z. (2015b). Ultra High Performance Liquid Chromatography with Electrospray Ionization Tandem Mass Spectrometry Coupled with Hierarchical Cluster Analysis to Evaluate Wikstroemia Indica (L.) C. A. Mey. From Different Geographical Regions. J. Sep. Sci. 38 (12), 2093–2100. 10.1002/jssc.201401398 25866087

[B79] Wei W. L. Huang L. F. (2015a). Simultaneous Determination of Ferulic Acid and Phthalides of Angelica Sinensis Based on UPLC-Q-TOF/MS. Molecules 20 (3), 4681–4694. 10.3390/molecules20034681 25781070 PMC6272380

[B80] Wen B. Y. Yan Y. Wang W. B. Wang Z. R. (2017). Pharmacokinetics of Effective Components after Oral Administration of Poria Triterpenoids Extract with Different Forms in Rats. Chin. J. Exp. Traditional Med. Formula 23, 97–101. 10.13422/j.cnki.syfjx.2017070097

[B81] Xia M.-C. Cai L. Xu F. Zhan Q. Feng J. Guo C. (2022). Whole-Body Chemical Imaging of Cordyceps Sinensis by TOF-SIMS to Visualize Spatial Differentiation of Ergosterol and Other Active Components. Microchem. J. 177, 107303. 10.1016/j.microc.2022.107303

[B82] Xia W. Hu S. Wang M. Xu F. Han L. Peng D. (2021). Exploration of the Potential Mechanism of the Tao Hong Si Wu Decoction for the Treatment of Postpartum Blood Stasis Based on Network Pharmacology and *In Vivo* Experimental Verification. J. Ethnopharmacol. 268, 113641. 10.1016/j.jep.2020.113641 33271240

[B83] Xu J. Liu J. Li B. Wei X. Qi Y. Zhang B. (2022). Comparison of Blood Tonic Efficacy and Chemical Constituents of Kadsura Interior A.C. Smith and its Closely Related Species. Chin. Med. 17 (1), 14. 10.1186/s13020-021-00544-w 35039063 PMC8762946

[B84] Yan G. Zou D. Zhang A. Tan Y. Sun H. Wang X. (2015). UPLC-Q-TOF-MS/MS Fingerprinting for Rapid Identification of the Chemical Constituents of Ermiao Wan. Anal. Methods 7 (3), 846–862. 10.1039/C4AY01215A

[B85] Yan Q. Mao H. Wei Y. (2017). Elucidation of Mechanism of Si-Jun-Zi Decoction-Induced Reversal of Spleen Deficiency Syndrome in Rats by LC-QTOF/MS Metabolomics. Trop. J. Pharm. Res. 16 (3), 525–533. 10.4314/tjpr.v16i3.5

[B86] Yang B. Li H. Ruan Q. F. Xue Y. Y. Cao D. Zhou X. H. (2018). A Facile and Selective Approach to the Qualitative and Quantitative Analysis of Triterpenoids and Phenylpropanoids by UPLC/Q-TOF-MS/MS for the Quality Control of Ilex Rotunda. J. Pharm. Biomed. Anal. 157, 44–58. 10.1016/j.jpba.2018.05.002 29758469

[B87] Yang B. Dong H. Sun H. Han Y. Zhang A. H. Yan G. L. (2019c). Urine Metabolomics Analysis of Toxicity Attenuation Effects of Fuzi Compatibility with Gancao. Mod. Chin. Med. 21 (07), 895–902. 10.13313/j.issn.1673-4890.20190702005

[B88] Yang C. Xia T. Wang C. Sun H. Li Y. Gong Z. (2019b). Using the UPLC-ESI-Q-TOF-MSE Method and Intestinal Bacteria for Metabolite Identification in the Nonpolysaccharide Fraction from Bletilla Striata. Biomed. Chromatogr. 33 (11), e4637. 10.1002/bmc.4637 31256429

[B89] Yang J. B. Liu Y. Wang Q. Ma S. C. Wang A. G. Cheng X. L. (2019a). Characterization and Identification of the Chemical Constituents of Polygonum Multiflorum Thunb. By High-Performance Liquid Chromatography Coupled with Ultraviolet Detection and Linear Ion Trap FT-ICR Hybrid Mass Spectrometry. J. Pharm. Biomed. Anal. 172, 149–166. 10.1016/j.jpba.2019.03.049 31048141

[B90] Yang M. Zhao Y. Qin Y. Xu R. Yang Z. Peng H. (2021b). Untargeted Metabolomics and Targeted Quantitative Analysis of Temporal and Spatial Variations in Specialized Metabolites Accumulation in Poria Cocos (Schw.) Wolf (Fushen). Front. Plant Sci. 12, 713490. 10.3389/fpls.2021.713490 34621284 PMC8490877

[B91] Yang S. Shan L. Luo H. Sheng X. Du J. Li Y. (2017a). Rapid Classification and Identification of Chemical Components of Schisandra Chinensis by Uplc-Q-Tof/ms Combined with Data Post-Processing. Molecules 22 (10), 1778–1796. 10.3390/molecules22101778 PMC6151474 29053630

[B92] Yang Z. Jiang M. Yue Z. Wang P. Wang H. Zhang G. (2021a). Metabonomics Analysis of Semen Euphorbiae and Semen Euphorbiae Pulveratum Using UPLC-Q‐Tof/MS. Biomed. Chromatogr. 36, e5279. 10.1002/bmc.5279 34783065

[B93] Yang Z. L. Zhao J. (2017b). Qualitative and Quantitative Analysis of Icariin Analogues in Epimedium Koreanum by UPLC-Q-TOF MS. J. Chin. Mass Spectrom. Soc. 38 (1), 19–29. 10.7538/zpxb.2017.38.01.0019

[B94] Yuan Q. Xie F. Tan J. Yuan Y. Mei H. Zheng Y. (2022). Extraction, Structure and Pharmacological Effects of the Polysaccharides from Cordyceps Sinensis: A Review. J. Funct. Foods 89, 104909. 10.1016/j.jff.2021.104909

[B95] Yuan R. Lin Y. (2000). Traditional Chinese Medicine: An Approach to Scientific Proof and Clinical Validation. Pharmacol. Ther. 86 (2), 191–198. 10.1016/S0163-7258(00)00039-5 10799714

[B96] Yuan T. Guo X.-F. Shao S.-Y. An R.-M. Wang J. Sun J. 2021). Characterization and Identification of Flavonoids from Bambusa Chungii Leaves Extract by UPLC-ESI-Q-TOF-MS/MS. Acta Chromatogr. 33(3), 281–294. 10.1556/1326.2020.00777

[B97] Zhang A. Sun H. Qiu S. Wang X. (2013). Advancing Drug Discovery and Development from Active Constituents of Yinchenhao Tang, a Famous Traditional Chinese Medicine Formula. Evid. Based Complement. Altern. Med. 2013, 257909. 10.1155/2013/257909 PMC3804150 24191164

[B98] Zhang B. Yu D. Luo N. Yang C. Zhu Y. (2020). Four Active Monomers from Moutan Cortex Exert Inhibitory Effects against Oxidative Stress by Activating Nrf2/Keap1 Signaling Pathway. Korean J. Physiol. Pharmacol. 24 (5), 373–384. 10.4196/kjpp.2020.24.5.373 32830144 PMC7445476

[B99] Zhang F. Zhan Q. Gao S. Dong X. Jiang B. Sun L. (2014). Chemical Profile- and Pharmacokinetics-Based Investigation of the Synergistic Property of Platycodonis Radix in Traditional Chinese Medicine Formula Shengxian Decoction. J. Ethnopharmacol. 152 (3), 497–507. 10.1016/j.jep.2014.01.033 24524880

[B100] Zhang X. Li P. Hua Y. Ji P. Yao W. Ma Q. (2018). Urinary Metabolomics Study the Mechanism of Taohong Siwu Decoction Intervention in Acute Blood Stasis Model Rats Based on Liquid Chromatography Coupled to Quadrupole Time-Of-Flight Mass Spectrometry. J. Chromatogr. B Anal. Technol. Biomed. Life Sci. 1074-1075, 51–60. 10.1016/j.jchromb.2017.12.035 29331744

[B101] Zhang Y. Feng B. M. Lv X. (2017). Research Progress on Application of UPLC/Q-TOF-MS in Pharmaceutical Analysis. Nat. Prod. Res. Dev. 29 (11), 1992–1996. 10.16333/j.1001-6880.2017.11.028

[B102] Zhao Q. L. Bian X. K. Qian D. W. Zhang T. Zhu Z. H. Guo S. (2019b). Comparative Study on Differences of Paeonia Lactiflora from Different Habitats Based on Fingerprint and Chemometrics. Zhongguo Zhong Yao Za Zhi 44 (15), 3316–3322. 10.19540/j.cnki.cjcmm.20190424.202 31602889

[B103] Zhao T. Tang H. Xie L. Zheng Y. Ma Z. Sun Q. (2019a2019). Scutellaria Baicalensis Georgi. (Lamiaceae): a Review of its Traditional Uses, Botany, Phytochemistry, Pharmacology and Toxicology. J. Pharm. Pharmacol. 71, 1353–1369. 10.1111/jphp.13129 31236960

[B104] Zhu F. Ruan L. Ma Y. Ji W. Liu H. (2014). Simultaneous Determination of 20 Illegally Added Anti-Diabetic Chemical Components in Hypoglycemic and Weight-Reducing Health Foods by Ultra-high Performance Liquid Chromatography-Tandem Mass Spectrometry]. Se Pu 32 (1), 13–20. 10.3724/sp.j.1123.2013.08035 24783863

[B105] Zhu S. S. Long R. Song T. Zhang L. Dai Y. L. Liu S. W. (2019). UPLC-Q-TOF/MS Based Metabolomics Approach to Study the Hepatotoxicity of Cantharidin on Mice. Chem. Res. Toxicol. 32 (11), 2204–2213. 10.1021/acs.chemrestox.9b00233 31617706

[B106] Zong X. X. Wang Y. Xiong J. L. Ping Y. H. Liang Q. L. (2022). Characterization of Alkaloids in Radix Sophora Tonkinensis by UPLC-Q-TOF-MS/MS and its Application in the Comparison of Two Different Habitats. Nat. Prod. Res. 36 (1), 429–431. 10.1080/14786419.2020.1771712 32468852

